# Symptoms induced by environmental irritants and health-related quality of life in patients with chronic cough - A cross-sectional study

**DOI:** 10.1186/1745-9974-7-6

**Published:** 2011-10-07

**Authors:** Ewa Ternesten-Hasséus, Sven Larsson, Eva Millqvist

**Affiliations:** 1 Department of Allergology, Institution of Internal Medicine, the Sahlgrenska Academy at University of Gothenburg, Sweden, S-413 45 Gothenburg, Sweden; 2 Department of Respiratory Medicine, Institution of Internal Medicine, the Sahlgrenska Academy at University of Gothenburg, Sweden

**Keywords:** Cough, environmental exposure, and quality of life

## Abstract

**Background:**

Chronic cough is a common condition, but some patients have no evident medical explanation for their symptoms. A group of patients has been identified, characterized by upper and lower airway symptoms triggered by chemicals and scents, and heightened cough sensitivity to inhaled capsaicin. Chronic cough is usually a prominent symptom in these patients, and it has been suggested that they suffer from sensory hyperreactivity.

Our main aim was to analyse, in a group of patients with chronic cough, the presence of symptoms induced by environmental factors such as chemicals, scents, and cold air, and to measure the social and emotional influences of these symptoms in relation to quality of life. A second aim was to pilot-test a Swedish translation of a cough-specific questionnaire.

**Methods:**

A total of 119 patients with chronic cough were asked to answer three different questionnaires: a local symptom questionnaire, the Chemical Sensitivity Scale for Sensory Hyperreactivity (CSS-SHR), and the Nottingham Health Profile (NHP). In addition, a Swedish version of the Hull Airway Reflux Questionnaire (HARQ) was developed and answered by a subgroup of patients and healthy controls.

**Results:**

Sixty-two patients (52%) with mean cough duration of 10.6 years answered the local symptom questionnaire, the CSS-SHR, and the NHP. Of these, 39 (63%) claimed to have cough and other symptoms induced by chemicals and scents. Compared to population-based findings, the patients scored higher on the CSS-SHR, and the CSS-SHR score was significantly higher among chemical-sensitive individuals (*p *< 0.001). The NHP showed that the patients had a significantly reduced quality of life, which was most pronounced among chemical-sensitive individuals. The 31 patients who answered the HARQ had significantly higher scores (*p *< 0.0001) than 59 healthy controls.

**Conclusions:**

Among patients with chronic cough, a majority claimed that environmental factors induced coughing. Both the CSS-SHR and the HARQ score systems seem to be valuable instruments in the mapping of cough patients, supporting the novel paradigm of a cough hypersensitivity syndrome. Our results emphasize that cough is a substantial burden to the patient, influencing daily living and quality of life.

## Background

Cough is an essential protective physiological mechanism to prevent food, liquid, dust, and chemicals from reaching the lower airways, but it is also a symptom of many inflammatory diseases of the lungs. Coughing is one of the commonest symptoms for which patients consult a doctor in the western world, and current therapies are often unsatisfactory [1]. Chronic cough is also clearly associated with significant social and psychological impacts [2,3]. Cough is arbitrarily defined as being chronic when it has lasted for more than 8 weeks [4], though the definition of chronic cough varies in the literature and the prevalence of this condition has been debated [4-6].

When known causes of cough, such as various infections, cancer, foreign body aspiration, cystic fibrosis, alveolitis, asthma, chronic obstructive pulmonary disease, medication with angiotensin-converting enzyme (ACE) inhibitor, gastro-oesophageal reflux disease (GERD), or post-nasal drip syndrome have been excluded, a group of patients with unexplained cough still remains. A discrete clinical entity has been suggested for patients with such chronic cough in combination with increased capsaicin cough sensitivity, in which the cough is often triggered by environmental stimuli, and furthermore, the cough initially developed after an upper respiratory tract infection [7]. Some cough patients can be labelled as having chronic refractory unexplained cough, a condition without medical explanation and persistent, ongoing coughing in case of thoroughly tries with different medications [6,8]. A similar group of patients has been identified, characterized by upper and lower airway symptoms triggered by chemicals and scents, and heightened cough sensitivity to inhaled capsaicin; it has been suggested that these patients suffer from sensory hyperreactivity (SHR) [9,10]. The Hull Airway Reflux Questionnaire (HARQ) was developed by Morice et al. to identify coughers, with a novel paradigm for understanding chronic cough [11]. This paradigm, the 'cough hypersensitivity syndrome', also includes patients with symptoms that may indicate a reflux disease, such as patients with a general hypersensitivity towards, for example, environmental irritants. The patients are classified as having a cough hypersensitivity syndrome that also comprises both sensitivity to environmental irritants and augmented capsaicin cough response [12-14].

The extent to which chronic cough is caused by perceived (hyper)sensitivity to chemicals and scents is not well known. Furthermore, we do not know whether the behavioural consequences and effects on health-related quality of life (HRQoL) are influenced by the presence of such hypersensitivity. These questions can, however, be analysed by applying established and local questionnaires.

The Chemical Sensitivity Scale for Sensory Hyperreactivity (CSS-SHR) was developed in order to quantify self-reported affective reactions to, and behavioural disruptions from, odorous/pungent substances [15]. It is useful for the diagnosis of SHR, and a high CSS-SHR score is directly related to capsaicin sensitivity [10].

The main aim of the present study was to analyse, in a group of patients with chronic cough without evident medical explanation, the presence of symptoms induced by environmental factors such as chemicals, scents, cold air, and exercise, and to measure the social and emotional influences of these symptoms in relation to quality of life. For this purpose a local questionnaire [16], the CSS-SHR [15], and the Nottingham Health Profile (NHP) [17] were used. A second aim was to pilot-test a Swedish translation of the cough-specific questionnaire HARQ [11].

## Methods

### Patients

The patients were selected from the medical records at the Department of Allergology and Respiratory Medicine at the Sahlgrenska University Hospital in Gothenburg, Sweden. Between January 2006 and December 2007, 479 patients attended the clinic due to chronic cough. From this group, 119 patients, aged 18 to 74 years (68 women; 57%), were invited to participate. The patients had originally been referred to the clinic due to at least two months of cough, and were diagnosed with chronic cough. Patients with pulmonary or other diseases that could cause cough were excluded, as were patients diagnosed with allergy, rhinitis, post-nasal drip, or any kind of GERD. Other exclusion criteria were any ACE inhibitors or medication for GERD, and current smoking.

### Study design

#### Part I

Three questionnaires (a local symptom questionnaire, the CSS-CHR, and the NHP) were sent by postal mail to all the patients, with a covering letter, informed consent form, and a prepaid return envelope. The estimated time to complete all three questionnaires was about 20 minutes. The patients were reminded once within a month, with a new letter and questionnaires. In some cases, after this, patients were phoned for complementary answers.

The patients were asked to answer the questions based on their experienced condition during the previous month.

#### Part II

A Swedish version of the HARQ was used as a pilot trial [11]. Thirty-one patients (21 women; 68%) who had participated in the first part of the study were consecutively asked to also answer the HARQ questionnaire. They were contacted by phone, and the questionnaire was then mailed with a prepaid return envelope.

A control group of 59 (39 women; 66%) consecutively selected, subjectively healthy, non-smoking individuals also answered the HARQ. They were screened using questions on airway symptoms, symptoms of GERD, and symptoms in response to chemicals, scents, cold air, and exercise; none of the control group had any of these symptoms. The controls were subject to no further medical examinations.

### Questionnaires

#### Local symptom questionnaire

The local symptom questionnaire contained questions concerning chemicals, scents, cold air, and exercise-induced symptoms (yes/no). Thirteen symptoms were analysed: cough, heavy breathing, difficulty getting air, chest pressure, phlegm, throat irritation, hoarseness, nasal blockage, rhinorrhoea, eye irritation, headache, dizziness, and fatigue. The participants were asked to evaluate symptoms on a scale of 0-3 (0, no symptoms; 1, mild; 2, moderate; and 3, severe symptoms) [16].

#### CSS-SHR

The CSS-SHR questionnaire was used to quantify the affective and behavioural consequences of self-reported sensitivity to chemicals and scents in the course of daily activities. The CSS-SHR is well validated and has a good reproducibility [15]. It consists of 11 statements/questions (see Additional file 1), selected from a large number of items for measuring odour intolerance on the basis of being particularly sensitive for discriminating patients with SHR from healthy controls [18]. The unweighted sum of all 11 items makes up the individual's total CSS-SHR score (0-54 points). A high score, ≥43 points, is regarded as a diagnostic cut-off value for SHR [15]. In a random general Swedish population of adults, the prevalence of such odour intolerance, defined as a CSS-SHR score ≥43, has been determined to be 19%, with an increased risk for female gender (odds ratio: 2.3) [19].

#### NHP

Health-related quality of life was assessed using the NHP questionnaire, which consists of two parts [17,20]. Norm and reference values distributed for age and sex based on data from larger population studies are used for both part I and part II [17,21,22].

Part I contains 38 items covering six aspects of HRQoL, concerning the domains of emotional reactions, sleep, energy, pain, physical mobility, and social isolation. The response alternatives for each item are 'yes' and 'no', depending on whether that item fits the individual's current situation. The answers are weighted for each dimension, giving a range from zero (no problems at all) to 100 (presence of all problems within the area) [23].

Part II of the NHP contains seven questions, again with yes/no alternatives, concerning the impact of health problems on the individual's social functioning in terms of paid employment, housework, social life, family life, sex life, hobbies, and holidays. The proportions of positive answers to each of the seven questions are calculated separately and compared with the reference values.

#### HARQ

The HARQ [11] was originally derived from the Reflux Symptom Index and modified to allow analysis of the symptomatology of reflux cough [24]. It was translated from English to Swedish by bilingual personnel, using a formal forward-backward translation, and two clinicians reviewed the questions. The back translation was almost identical to the source document. A preliminary Swedish version was then determined. The HARQ is a self-administered instrument, and consists of 14 items (see Additional file 2). The participants were asked to evaluate how different problems had affected them during the previous month, on a scale of 0-5 (0, no problem; 5, severe/frequent problems), with the total score varying from 0−70 points and an upper normal total score limit of 13 points.

### Statistical methods

All data were analysed using version 17 of the SPSS software package (SPSS, Inc., Chicago, IL, USA). Data are presented as mean values with standard deviation (SD), and a p value of < 0.05 was taken for statistical significance. For the local symptom questionnaire, the CSS-SHR, and the HARQ, an independent t-test and the Mann-Whitney *U*-test were applied for unpaired data, and the chi-square test for categorical data. For the NHP, the unpaired data in part I were analysed with the Mann-Whitney *U*-test, and the chi-square test was used in part II.

The ability of the HARQ to distinguish patients from healthy controls was evaluated by constructing a receiver operating characteristic (ROC) curve, and plotting the sensitivity versus 1-specificity for each possible cut-off score [25]. An area under the curve of more than 0.90 indicates that a method has outstanding discrimination ability, for example the ability to distinguish two groups from each other [26].

Informed consent was obtained from all participants at the start of the investigation. The study was approved by the Regional Ethical Review Board of Gothenburg, Sweden.

## Results

### Part I

**Figure 1:**
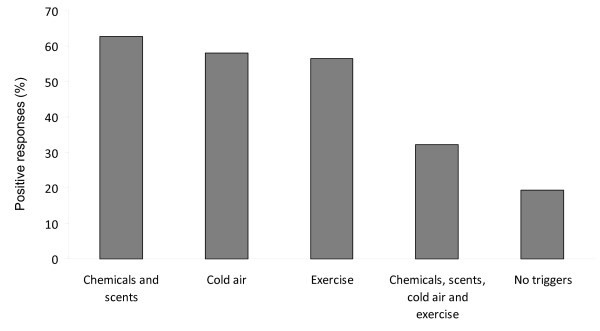
**Percentage of positive responses of reported trigger factors in 62 patients with chronic cough**.

Seventy out of 119 patients completed all questionnaires, giving a response rate of 59%. Six patients were excluded from the analysis due to having recovered, and two due to current smoking, leaving a total of 62 participants (52%). Demographic data of the study group are shown in table 1 and use of cough medication in table 2. All ex-smokers had stopped smoking more than one year earlier. Percentage distributions of reported trigger factors are shown in figure 1. There were no differences in reported trigger factors between men and women.

**Table 1:** Demographic data for 62 patients with chronic cough

Characteristics	Subjects (n = 62)
Sex, female/male (n)	40/22
Age (years)	53.6 (11.9)
Duration of cough symptoms (years)	10.6 (10.0)
FEV_1 _% predicted	103.8 (16.9)
BMI (kg/m^2^)	25.1 (4.8)
**Smoking status (n)** Never/previous	38/24
**Chest X-ray (n)** Normal/unknown	59/3
**Methacholine provocation test (n)** Normal/unknown	52/10

Data are shown as mean and standard deviation (SD) for age, duration of cough symptoms, FEV_1_, BMI, and otherwise as number (n).

*Definition of abbreviations*: FEV_1 _= Forced expiratory volume in one second; BMI = Body mass index.

**Table 2:** Current or earlier use of medication for coughing in 62 patients with chronic cough

Medication	Number of users (% of the whole group)	Some effect Number (% of users)
Oral steroids	8 (13)	0
Inhaled corticosteroids	31 (50)	4 (13)
Inhaled β_2 _agonist	27 (44)	4 (15)
Inhaled anticholinergics	9 (15)	1 (11)
Morphine derivate syrup	16 (26)	13 (81)
Codeine	3 (5)	3 (100)

#### Local symptom questionnaire

**Figure 2:**
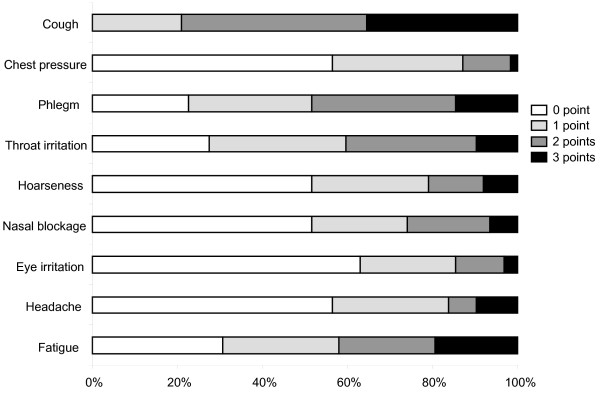
**Evaluation of nine symptoms on a scale of 0-3 (0, no symptoms; 1, mild; 2, moderate; and 3, severe symptoms) in 62 patients with chronic cough**.

Except for cough, the most common symptoms and symptoms with the highest scores were phlegm, throat irritation, and fatigue. The patients' symptom scores are shown in figure 2.

Women reported more problems with eye irritation (p < 0.05) compared to men, but otherwise there were no differences in symptoms between men and women.

Thirty-nine patients (63%; 27 women and 12 men [*ns*]) claimed to have symptoms induced by chemical and scent exposure. In the present article, these 39 are defined as the chemical-sensitive group and patients without chemical sensitivity as the non-sensitive group.

The chemical-sensitive group reported more problems with heavy breathing (p < 0.01), difficulty getting air (p < 0.01), chest pressure (p < 0.01), nasal blockage (p < 0.05), eye irritation (p < 0.01), headache (p < 0.01), dizziness (p < 0.01), and fatigue (p < 0.001) compared to the non-sensitive group.

#### CSS-SHR

The mean (SD) CSS-SHR score for the whole group was 38.7 (9.6). There were no differences in the scores between men and women. The mean CSS-SHR score was 43.1 (6.5) in the chemical-sensitive group compared to 31.2 (9.5) in the non-sensitive group (p < 0.001).

Patients were divided into two groups according to the cut-off value for CSS-SHR [15]: those with scores ≥43 points (high score) and those with scores < 43 points (low score). Twenty-four patients (39%; 15 women) had a score of ≥43 points. Of these, 21 belonged to the chemical-sensitive group.

#### NHP

In part I of the NHP, the patients differed significantly from the reference group in the areas of sleep (p < 0.01), energy (p < 0.05), pain (p < 0.001), physical mobility (p < 0.001), and social isolation (p < 0.001). The chemical-sensitive group had more problems in the areas of energy (p < 0.01), pain (p < 0.01), and physical mobility (p < 0.001) compared to the non-sensitive group. Patients with high CSS-SHR score experienced more HRQoL problems with energy (p < 0.05), pain (p < 0.001), and physical mobility (p < 0.05), compared to the low-scoring group.

**Figure 3:**
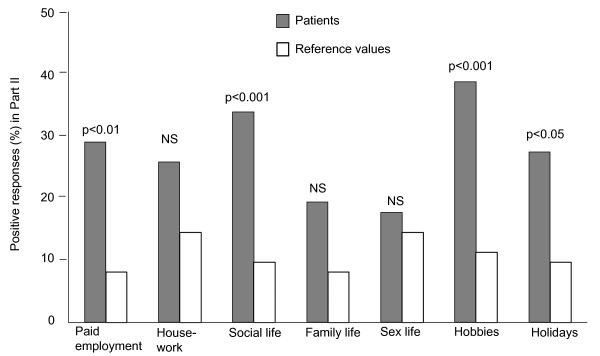
**Percentage of positive responses reflecting problems in daily activities in part II of the NHP for 62 patients with chronic cough (filled columns) and reference values (open columns)**. NS = not significant.

In part II, the patients differed significantly from the reference group in four of seven areas (Figure 3). The chemical-sensitive group had more problems than the non-sensitive group in five of seven areas: paid employment (p < 0.01), housework (p < 0.01), social life (p < 0.01), sex life (p < 0.05), and holidays (p < 0.05). Patients with high CSS-SHR score experienced more HRQoL problems than the low-scoring group in five of seven areas: paid employment (p < 0.01), housework (p < 0.05), sex life (p < 0.05), hobbies (p < 0.05), and holidays (p < 0.01).

There were no differences in HRQoL between genders.

### Part II

#### HARQ

Fully completed questionnaires were obtained from 31 patients and 59 healthy controls. The two groups were matched by gender, but not by age; mean age was 52.6 (12.8) years in the patient group, and 45.0 (14.7) years among the controls (p < 0.01).

**Figure 4:**
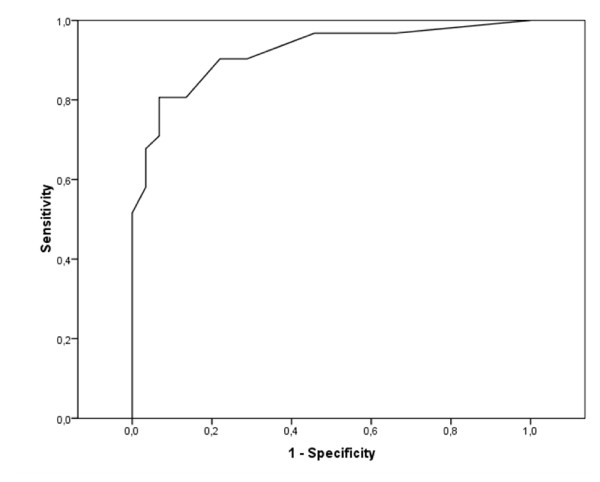
**ROC curve of ability to distinguish patients with chronic cough from healthy controls**.

The mean total HARQ score was 17.0 (11.2) in the patient group and 2.2 (3.0) among the controls (p < 0.0001). The cut-off limit of 13 points was exceeded by 16 patients (52%; 12 women), but in none of the healthy controls. The area under the ROC curve was 0.92, which corresponds to outstanding discrimination ability (Figure 4).

Twenty-two patients (71%; 15 women) belonged to the chemical-sensitive group. There were no differences in HARQ score between these patients and the non-sensitive group. Thirteen patients (42%; 9 women) belonged to the group scoring high on the CSS-SHR. These patients reported more problems with 'cough with certain food' compared to the patients with low CSS-SHR scores (p < 0.05).

Among the patients, the mean total score was 20.2 (10.0) for women and 11.0 (11.0) for men (p < 0.05). The women also had significantly higher scores for 'excess mucus in the throat, or drip down the back of your nose' (p < 0.05), 'heartburn, indigestion, stomach acid coming up' (p < 0.05), 'cough brought out by singing or speaking' (p < 0.05), and 'a strange taste in your mouth' (p < 0.05) compared to men. There were no gender differences among the healthy controls.

## Discussion

The main findings in this study were that more than 60% of the patients with chronic cough reported that coughing could be induced by environmental factors like chemicals, scents, exercise, and cold air, and that this coughing had a negative impact on the patients' health status and daily lives. The patients had increased CSS-SHR and NHP scores compared to population-based findings, and higher total HARQ score compared to healthy controls.

About 40% of the chronic cough patients in this study had a high CSS-SHR score (≥43 points), exceeding the cut-off limit, which can be compared with a corresponding value of 19% in a population-based study [19]. These results indicate that SHR could be one explanation for the reported symptoms. However, a standardized capsaicin inhalation test would be required to diagnose whether these patients actually did have SHR, and such a study is planned. Earlier studies confirm that many patients with SHR have symptoms similar to those of patients with chronic cough [27-30].

The cough patients with chemical sensitivity reported significantly more symptoms from the eyes, nose, and lungs, as well as headache and fatigue, which is in accordance with earlier results [19]. These findings, together with the current results of impaired HRQoL among chemical-sensitive and high CSS-SHR scoring individuals, may indicate a more troublesome form of cough. The results can be compared with a recent study reporting a median CSS-SHR score of 35 in a group of asthma patients before treatment with inhaled corticosteroids, in comparison to a score value of 25 in the control group [31]. It is not known whether increased CSS-SHR scores are also seen in other chronic conditions like irritable bowl syndrome and chronic pain, but it would be of interest to study this in the future.

There were no differences in the CSS-SHR scores between men and women, and women were not more common in the high-scoring group, though women did report more symptoms from the eyes compared to men. These results contradict earlier studies of the Swedish population, and on SHR, showing a dominance of women with high CSS-SHR scores [15,19].

The chronic cough patients in the present study had significantly impaired HRQoL compared with a population sample, as assessed by the generic instrument NHP, and also in comparison with other chronic conditions tested with the NHP [32-34]. The findings are in accordance with earlier studies in patients with SHR, also assessed with the NHP [16,35]. There were no gender differences in HRQoL; this is in contrast to other studies of patients with chronic cough, which have shown women to be more adversely affected than men regarding HRQoL in several dimensions of daily life [36].

The preliminary Swedish version of the HARQ has not yet been validated, and so cannot be used to draw any major conclusions, though we hypothesise that the Swedish translation will give results in concordance with the original British version. However, the high HARQ scores in the present study are striking, especially considering that the questionnaire was originally constructed for patients with cough and GERD, and patients with any history of reflux symptoms or use of medication for such condition were excluded from this study. This implies that the HARQ mirrors not only the symptoms of cough caused by GERD, but also a hypersensitivity cough syndrome with other causes, in agreement with earlier suggestions [11-13]. In accordance with Morice et al., women with chronic cough had significantly higher total scores compared to men, and the area under the ROC showed outstanding discrimination ability to distinguish patients from healthy controls [11]. In future studies it will be crucial to make a formal validation of the Swedish version of the HARQ, assessing repeatability and validity.

Six patients were excluded from the data analysis because they had recovered from their cough. Interestingly, none of these patients reported airway sensitivity to chemicals and scents. However, their number is too small to allow any major conclusions as to whether chemical-induced symptoms could be an important factor for developing persistent cough. A longitudinal study revealed that even after five years, patients with cough and other airway symptoms induced by chemicals and scents had lasting symptoms, a reduced quality of life, and unchanged sensory hyperreactivity, implying that the condition could be regarded as chronic [16]. One challenge for the future will be to study whether chemical sensitivity is crucial to the disease duration in a larger group of patients with chronic cough.

In recent years there has been emerging interest in the family of transient receptor potential (TRP) ion channels. These proteins are able to sense temperature, noxious stimuli, pain, stretch, and osmolarity, among other factors. The main foci of such triggers in the airways are ion channels belonging to the transient receptor potential vanilloid (TRPV) and the transient receptor potential ankyrin families [37-39]. Nociceptive sensory neurons also participate in protective reflexes, including the cough and sneeze reflexes, and release inflammatory neuropeptides in the periphery upon stimulation by various environmental stimuli. Patients with chronic cough have been shown to have an increase in the transient receptor potential vanilloid type 1 (TRPV1) staining nerve profiles, and also a significant correlation between capsaicin tussive response and the number of TRPV1-positive nerves [40,41]. Several studies have shown that patients with chronic cough have increased capsaicin sensitivity [12,14]. The results from all these studies suggest that the pathophysiology of chronic cough is related to airway mucosal TRP receptors on sensory nerves, as well as reaction to noxious stimuli [38]. This is in line with the present finding that environmental triggers such as chemicals and scents constitute a major cough trigger. The related ion channel, the transient receptor potential melastin type 8 (TRPM8), which is activated by menthol and cold air, could be part of an explanation as to why a majority of the study patients also reported cold air and exercise as cough-inducing factors [42]. These results are also in accordance with a recent study showing coughing and increased cough sensitivity in patients with SHR after provocations with exercise in cold air [30].

In summary, our results underline the importance of widening the views regarding chronic cough. Both the CSS-SHR and the HARQ score system turned out to be valuable instruments in the mapping of cough patients, supporting the novel paradigm of a cough hypersensitivity syndrome. The study also once more emphasizes that cough is a substantial burden to the patient, influencing daily life and HRQoL.

## Abbreviations

ACE: angiotensin-converting enzyme; BMI: body mass index; CSS-SHR: chemical sensitivity scale for sensory hyperreactivity; FEV_1_: forced expiratory volume in one second; GERD: gastro-oesophageal reflux disease; HARQ: Hull Airway Reflux Questionnaire; HRQoL: health-related quality of life; NHP: Nottingham Health Profile; NS: not significant; SD: standard deviation; SHR: sensory hyperreactivity; TRP: transient receptor potential; TRPM8: transient receptor potential melastin type 8; TRPV: transient receptor potential vanilloid; TRPV1: transient receptor potential vanilloid type 1.

## Competing interests

The authors declare that they have no competing interests.

## Authors' contributions

All the authors participated in the design of the study and the study questionnaires. ETH coordinated and analysed data from the returned questionnaires and drafted the manuscript.

EM and SL helped to draft the manuscript. All authors read and approved the final manuscript.

## Supplementary Material

Additional file 1 **Items of the Chemical Sensitivity Scale for Sensory Hyperreactivity (CSS-SHR)**. Click here for file

Additional file 2 **Items of the Hull Airway Reflux Questionnaire (HARQ)**. Click here for file
