# Supplementary material for: Symptoms induced by environmental irritants and health-related quality of life in patients with chronic cough - A cross-sectional study
Source: Cough. 2011 Oct 7;7:6. doi: 10.1186/1745-9974-7-6 (PMC3212888; doi:10.1186/1745-9974-7-6)
Supplement: Additional file 1 — Items of the Chemical Sensitivity Scale for Sensory Hyperreactivity (CSS-SHR). [file 1745-9974-7-6-S1.PDF]

Additional File 1

Items of the Chemical Sensitivity Scale for Sensory Hyperreactivity (CSS-SHR)

A. I would not mind living on a street with odorous/pungent car exhausts, if the apartment I had was nice[1]

B. I am more aware of odorous/pungent substances than I used to be[1,2]

C. At movies, other persons' perfume and after shave disturb me[1,2]

D. I am easily alerted by odorous/pungent substances[1,2]

E. I get used to most odorous/pungent substances without much difficulty[1]

F. How much would it matter to you if an apartment you were interested in renting were located close to a factory that emits odorous/pungent substances? [2,3]

G. In public places, I do not mind some smell of cigarette smoke[1]

H. There are often times when I want a complete odour-free environment[1,2]

I. I find it hard to relax in a place that evokes odorous/pungent sensations.[2,4]

J. I would not mind living in an apartment that has a weak smell[1]

K. I am sensitive to odorous/pungent substances[1,2]

---

[1]Scale: Agree strongly (0), agree (1), agree mildly (2), disagree mildly (3), disagree (4), disagree strongly (5). The numbers in parentheses refer to the score given for that response.

[2]Item scored in opposite direction before responses are summed.

[3]Scale: It would completely deter me (0), or it would be very important (1), important (2), slightly important (3), or not important at all (4).

[4]Scale: Always (0), very often (1), often (2), occasionally (3), seldom (4), never (5).
