# Supplementary material for: Symptoms induced by environmental irritants and health-related quality of life in patients with chronic cough - A cross-sectional study
Source: Cough. 2011 Oct 7;7:6. doi: 10.1186/1745-9974-7-6 (PMC3212888; doi:10.1186/1745-9974-7-6)
Supplement: Additional file 2 — Items of the Hull Airway Reflux Questionnaire (HARQ). [file 1745-9974-7-6-S2.PDF]

Additional file 2

Items of the Hull Airway Reflux Questionnaire (HARQ)

Within the last month, how did the following problems affect you?

A. Hoarseness or a problem with your voice[1]

B. Clearing your throat[1]

C. Excess mucus in the throat, or drip down the back of your nose[1]

D. Retching or vomiting when you cough [1]

E. Cough on first lying down or bending over[1]

F. Chest tightness or wheeze when coughing[1]

G. Heartburn, indigestion, stomach acid coming up (or do you take medications for this, if yes score 5)[1]

H. A tickle in your throat, or a lump in your throat[1]

I. Cough with eating (during or soon after meals)[1]

J. Cough with certain foods[1]

K. Cough when you get out of bed in the morning[1]

L. Cough brought on by singing or speaking (for example, on the telephone)[1]

M. Coughing during the day rather than the night[1]

N. A strange taste in your mouth[1]

---

[1] Scale: 0 to 5, 0 = no problem and 5 = severe/frequent problem
